# Suppressive Effects of Kouboku on Methyl Mercaptan Production and Biofilm Formation in *Porphyromonas gingivalis*

**DOI:** 10.1111/omi.12493

**Published:** 2025-02-27

**Authors:** Yuri Taniguchi, Kazuhisa Ouhara, Yoko Sato, Mikio Shoji, Yitong Hou, Ruoqi Zhai, Ryousuke Fujimori, Naoya Kuwahara, Tetsuya Tamura, Shinji Matsuda, Noriyoshi Mizuno

**Affiliations:** ^1^ Department of Periodontal Medicine, Graduate School of Biomedical and Health Sciences Hiroshima University Hiroshima Japan; ^2^ Department of Microbiology and Oral Infection, Graduate School of Biomedical Sciences Nagasaki University Nagasaki Japan; ^3^ Department of Public Oral Health, Program of Oral Health Sciences, Graduate School of Biomedical and Health Sciences Hiroshima University Hiroshima Japan

**Keywords:** halitosis, Kouboku, periodontitis, porphyromonas gingivalis

## Abstract

*Porphyromonas gingivalis*, the bacterium responsible for periodontitis, produces several pathogenic factors, including methyl mercaptan, which contribute to the disease. Kouboku (Magnoliaceae), a Chinese herbal medicine, has been shown to suppress methyl mercaptan production from *P. gingivalis*. In this study, we investigated the inhibitory effect of Kouboku on methyl mercaptan production, biofilm formation, *P. gingivalis*‐host cell interactions, and its potential synergistic antibacterial effect with antibiotics. Five standard and five clinically isolated *P. gingivalis* strains were evaluated. Methyl mercaptan production was measured using OralChroma. The mRNA expression of *mgl* and *fimA*, which are involved in methyl mercaptan synthesis and adhesion molecules, was assessed using quantitative PCR. Biofilm formation by *P. gingivalis* and epithelial cell adhesion were analyzed following treatment with or without Kouboku. Furthermore, the effects of the active ingredients of Kouboku, honokiol, and magnolol, on the minimum inhibitory concentrations (MICs) of antibiotics against *P. gingivalis* were determined. No significant differences were observed in the suppression of methyl mercaptan production among *P. gingivalis* strains with different FimA genotypes treated with Kouboku. Moreover, Kouboku inhibited biofilm formation in co‐cultures of *P. gingivalis* and *Fusobacterium nucleatum*, as well as the adhesion of *P. gingivalis* to gingival epithelial cells through the downregulation of *fimA*. Treatment with honokiol and magnolol reduced the MICs of ampicillin, gentamicin, erythromycin, and tetracycline against *P. gingivalis*. These findings demonstrate that Kouboku affects *P. gingivalis* by modulating its adhesion to other bacteria and host cells, and enhances the antibacterial activity of certain antibiotics.

## 1 Introduction

Periodontitis is a periodontal disease involving inflammation of the periodontal tissue (Socransky and Haffajee 1993). Symptoms of periodontal disease include bad breath, swollen and painful gums, and tooth loss. Halitosis affects many people (Scully and Rosenberg 2003). Volatile sulfur compounds (VSCs) such as hydrogen sulfide, methyl mercaptan, and dimethyl sulfide cause halitosis (Kleinberg and Westbay 1990). The severity of periodontal disease has been found to be proportional to the amount of VSCs produced during halitosis (Morita and Wang 2001; Scully and Rosenberg 2003). Among VSCs, methyl mercaptan is most strongly detected in the oral cavity of patients with periodontal disease because of its strong production capacity by *Porphyromonas gingivalis* bacteria in the red complex, which is believed to be involved in the development and progression of periodontal disease (Ratcliff and Johnson 1999). It is also believed that methyl mercaptan is produced by the action of *P. gingivalis* bacteria on the saliva and detached epithelial cells in the oral cavity (Nakano et al. 2002). Locally, *P. gingivalis* produces pathogenic factors, including outer membrane proteins (Omps), gingipain, and proteolytic enzymes to generate VSCs (Liu et al. 2013; Yoshimura et al. 2000). l‐methionine‐α‐deamino‐γ‐mercaptomethane lyase (METase) produces ammonia and methyl mercaptan from l‐cysteine, whereas l‐cysteine‐desulfhydrase acts on l‐cysteine to produce pyruvate, ammonia, and hydrogen sulfide (Bollen et al. 1999). A previous study demonstrated that *P. gingivalis* produces large amounts of methyl mercaptan via METase encoded by *mgl* (Yoshimura et al. 2000). Furthermore, intraperitoneal injection of *mgl*‐deficient *P. gingivalis* mutants into mice led to a higher survival rate than an injection of wild‐type *P. gingivalis* (Yoshimura et al. 2000). These findings indicate that *mgl* may not only be involved in the production of methyl mercaptan but may also be associated with the virulence of *P. gingivalis*.

Treatment of halitosis associated with periodontal disease often involves visits to the dentist's office and patient brushing. However, in today's aging society, the number of patients who are unable to brush themselves or visit dentists continues to increase. Therefore, more convenient oral care methods are required. We hypothesized that it would be beneficial if patients who had difficulty brushing and gargling could be treated with a drink; therefore, we investigated the Chinese herbal medicine Kouboku. It is known to have therapeutic effects on gastrointestinal disorders, such as suppression of gastric juice secretion, protection of the gastric mucosa, and antiemetic and antibacterial activities (Munroe et al. 2007). In a previous study, the expression of *mgl* mRNA by *P. gingivalis* was strongly correlated with the production of methyl mercaptan in patients with halitosis. Kouboku also inhibits the production of methyl mercaptan and suppresses the mRNA expression of *mgl* (Ouhara et al. 2015). However, the mechanism by which the antimicrobial action of Kouboku affects periodontal disease remains largely unknown. Therefore, in this study, we aimed to determine the effects of Kouboku on the production of methyl mercaptan and *P. gingivalis* virulence factors.

## 2 Materials and Methods

### 2.1 Reagents

Kouboku (Magnoliaceae) was purchased from Sigma‐Aldrich (L9634, St. Louis, MO, USA). It was dissolved in phosphate‐buffered saline (PBS) before being used in in vitro and in vivo experiments. Honokiol and magnolol were purchased from FUJIFILM Wako Pure Chemical Corporation (Tokyo, Japan). They were dissolved in methanol and used in in vitro experiments.

### 2.2 Bacterial Strains

The standard bacterial strains used in this study were purchased from the American Type Culture Collection (ATCC) or kindly provided by Professor Atsuo Amano (Osaka University). Clinical isolates of *P. gingivalis* were used as previously reported (Ouhara et al. 2015)*. P. gingivalis* and *Fusobacterium nucleatum* were cultured on a sheep blood agar plate using an Anaeropack system (Mitsubishi Gas Chemical, Tokyo, Japan) at 37°C. After two days of incubation, the bacteria were inoculated into 40 mL of trypticase soy broth supplemented with 1% yeast extract, hemin (200 µg), and menadione (20 µg) using an Anaeropack system at 37°C. Finally, the bacteria were harvested during the exponential growth phase and washed with PBS. The *P. gingivalis mgl* knockout mutant was constructed using a double recombination strategy with the insertion of an *ermF* cassette, as described in a previous study (a protein secretion system linked to Bacteroidete gliding motility and pathogenesis) (Sato et al. 2010). The targeting DNA was prepared as follows: the 0.5 kb upstream and downstream regions of *mgl* were amplified using two pairs of primers and the genome of ATCC 33277 as a template. The *ermF* cassette was amplified using primers ermF‐F: [ATATCAAACAGGATCCCCCGATAGCTTCCG] and ermF‐R: [ATAAGGCCCCGGATCCCCTACGAAGGATGA] with the genome of the *gtfF (PGN_1668)::ermF* mutant (KDP611) as a template. The three purified PCR products were combined and used as templates for amplification. The final product was introduced into *P. gingivalis* ATCC 33277 via electroporation and transformants were selected on erythromycin‐supplemented blood agar plates (10 µg/mL). Proper mutation of *mgl* was confirmed using PCR.

### 2.3 Culture of OBA‐9 Cells

Human gingival epithelial cells (HGECs, #PCS‐200‐014) were purchased from the ATCC. The cells were subcultured in Dermal Cell Basal Medium (PCS‐200‐030, ATCC) supplemented with a Keratinocyte Growth Kit (PCS‐200‐040, ATCC) and 100 U penicillin/streptomycin (P4333‐100ML, Sigma‐Aldrich) at 37°C in a humidified atmosphere with 5% CO_2_. Confluent cultures of HGECs were used in subsequent experiments.

### 2.4 mRNA Expression of *mgl* and *fimA* in *P. gingivalis*

An overnight culture of *P. gingivalis* was pelleted by centrifugation (3000 × *g* for 10 min). The pellet was washed and resuspended in PBS, and the bacterial suspension was adjusted to a density of 1 × 10⁸ bacterial cells/mL. Kouboku (0, 10, 50, or 100 µg/mL) was then added to the bacterial suspension. The samples were then transferred to 1.5‐mL tubes and incubated for 10 min at 37°C. After treatment, the bacterial pellet was used to analyze the expression of *mgl* and *fimA* mRNA. Briefly, total RNA was extracted from *P. gingivalis* using RNAiso (Takara, Shiga, Japan), according to the manufacturer's protocol. Total RNA was purified via DNase I treatment and analyzed using a two‐step real‐time PCR system (Ouhara et al. 2015). Briefly, 1 µg of total RNA was reverse‐transcribed using ReverTra Ace qPCR RT Master Mix (TOYOBO, Tokyo, Japan). One microliter of cDNA was used for qPCR analysis. The amplification conditions were as previously described (Ouhara et al. 2015). qPCR was performed using the StepOnePlus software (Applied Biosystems, Foster City, CA, USA). Template cDNA (1 µL) was mixed with Core Reagent Fast SYBR Master Mix system (4 µL; Applied Biosystems), distilled water (4.5 µL), and primers (0.5 µL). The following primer sets were used for quantitative PCR: 16S rRNA forward, 5′‐TGTAGATGACTGATGGTGAAAACC‐3′ and 16S rRNA reverse, 5′‐TTTAGAGATTCGCATCCGGT‐3′; *mgl* forward, 5′‐TTCCGAGC TTCCCCCAATAC‐3′ and *mgl* reverse, 5′‐ATGAGGGTTTCCGTATCGCC‐3′) and *fimA* forward, 5′‐CTGTGTGTT TATGGCAAACTTC‐3′ and *fimA* reverse, 5′‐AACCCCGCTCCCTGTATTCCGA‐3′).

### 2.5 Measurement of Methyl Mercaptan Levels in *P. gingivalis*

To measure methyl mercaptan production by *P. gingivalis* in vitro, methyl mercaptan was synthesized using a previously described method (Yoshimura et al. 2000). Briefly, bacterial strains were grown at 37°C until the optical density at 660 nm (OD_660_) reached 1.0. Bacterial cells were harvested and washed with a buffered salt solution (40 mM potassium phosphate buffer [pH 7.7] and 50 mM sodium chloride). The cells were resuspended in this salt solution to an OD_660_ of 1.0. To quantify the production of methyl mercaptan, a reaction mixture consisting of 100 µL of the cell suspension and 870 µL of the buffered salt solution was added to a sterile 1.5 mL polypropylene tube sealed with a silicon plug. The reaction was initiated by adding 30 µL of 33 mM l‐methionine (to a final concentration of 10 mM l‐methionine). We then added Kouboku (0, 0.1, 1, 10, 50, 100 µg/mL) to the reaction mixtures and incubated them at 37°C. After 60 min, the reaction was stopped by the addition of 500 µL of 3 M phosphoric acid. A sample (1 mL) of the vapor above the reaction mixture in the tube was removed using a gastight syringe and analyzed using OralChroma (Nissha FIS, Inc., Tokyo, Japan).

### 2.6 Biofilm Formation Assay

Overnight cultures of *P. gingivalis* and *F. nucleatum* were pelleted by centrifugation (3000 × *g* for 10 min). The pellet was then washed and resuspended in PBS. The bacterial suspension was adjusted to a density of 10^8^ bacterial cells/mL (*P. gingivalis*: *F. nucleatum* = 5:1), and Kouboku (0, 0.1, 1, 10, 100 µg/mL) and l‐methionine (1 mM) were added to the bacterial suspension. The samples were cultured in 96‐well plates and incubated for 2 days at 37°C. After washing the plate once with PBS, the biofilm was stained with 1% crystal violet and then washed twice with PBS. Crystal violet was eluted from the plate using ethanol, and absorbance was measured at 450 nm using an ELISA reader (Varioskan LUX, Thermo Fisher Scientific, Waltham, MA, USA). To quantify biofilm formation, the ratio of biofilm formation was calculated for each sample and expressed as a ratio relative to the 0 µg/mL Kouboku group (no stimulation).

### 2.7 Adhesion Assay of *P. gingivalis* to OBA‐9 Cells

An overnight culture of *P. gingivalis* was pelleted by centrifugation (3000 × *g* for 10 min). The pellet was then washed and resuspended in PBS. The bacterial suspension was adjusted to a density of 1 × 10^7^ bacterial cells/mL, and Kouboku (0, 0.1, 1, 10, and 100 µg/mL) was added to the bacterial suspension. The samples (100 µL) were incubated for 10 min at 37°C, added to each well of a 96‐well plate containing OBA‐9 cells (1 × 10^5^ cells/well), and incubated for 2 h at 37°C. Samples cultured in 96‐well plates were washed thrice with PBS, and 0.25% trypsin was added to remove adherent bacterial cells from the plastic dish. After adding 100 µL PBS, the bacterial suspension was diluted appropriately, plated on a sheep blood agar plate, and incubated for 3 days at 37°C. The colonies were counted as adhesive bacterial cells.

### 2.8 Minimum Inhibitory Concentration (MIC) Measurement

Two‐day cultures of *P. gingivalis* were prediluted to an OD_660_ of 0.1, and 10 µL of the diluted culture was added to a 96‐well plate containing the appropriate culture medium. The plate was then incubated for 3 days at 37°C under anaerobic conditions. To measure the MIC, the culture medium containing the corresponding antibiotic solutions and magnolol or honokiol was serially 1:2‐diluted to final concentrations of 1024 to 0.03 µg/mL. MIC was determined visually as the lack of turbidity after 72 h.

### 2.9 Statistical Analysis

All experiments were performed independently at least three times. The Shapiro–Wilk test for distribution normality was conducted for each dataset. Data are expressed as mean ± standard deviation. Comparisons between the two groups were performed using two‐tailed unpaired Student's *t‐*test. *p*‐values < 0.05 were considered statistically significant.

## 3 Results

### 3.1 Methyl Mercaptan Production in *P. gingivalis*

**FIGURE 1:**
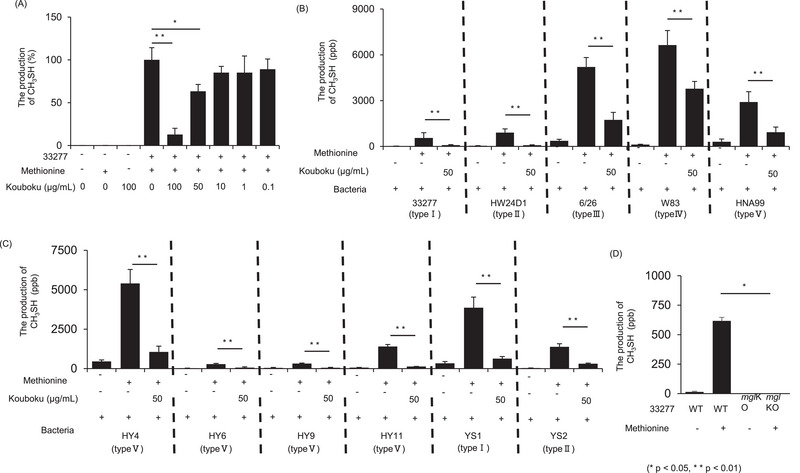
Methyl mercaptan production in *Porphyromonas gingivalis*. Methyl mercaptan production by *P. gingivalis* was measured using an OralChroma assay. (A) Production of methyl mercaptan by *P. gingivalis* 33277 in the presence or absence of Kouboku as measured using an OralChroma assay. *P. gingivalis* cells of different FimA genotypes were investigated. Representative standard strains (B) and clinical isolates (C) of *P. gingivalis*. Methyl mercaptan production by P. gingivalis 33277 wild‐type and mgl deletion mutants was measured using an OralChroma assay. (D) Bacterial strains were grown at 37°C until their optical density at 660 nm (OD_660_) reached 1.0. Bacterial cells were harvested and washed with a buffered salt solution (40 mM potassium phosphate buffer [pH 7.7] and 50 mM sodium chloride). The cells were resuspended in this salt solution to an OD_660_ of 1.0. To quantify methyl mercaptan production, a reaction mixture consisting of 100 µL of the cell suspension and 870 µL of the buffered salt solution was added to a sterile 1.5 mL polypropylene tube sealed with a silicon plug. The reaction was initiated by adding 30 µL of 33 mM l‐methionine (to a final concentration of 10 mM l‐methionine). Kouboku (0, 0.1, 1, 10, 50, or 100 µg/mL) was then added to the reaction mixtures and incubated at 37°C. After 60 min of incubation, the reaction was stopped by the addition of 500 µL of 3 M phosphoric acid. A sample (1 mL) of the vapor above the reaction mixture in the tube was removed using a gastight syringe and analyzed using OralChroma. *p < 0.05, **p < 0.01; Student's *t*‐test.

Measurement of the OralChroma data showed that Kouboku inhibited the production of methyl mercaptan in a concentration‐dependent manner (Figure 1A). Specifically, the addition of Kouboku (100 µg/mL) suppressed methyl mercaptan production by 87.1% (Figure 1A).

Previous studies have shown that *mgl* expression and methyl mercaptan production differ among strains (Ouhara et al. 2015). Thus, we investigated whether Kouboku differentially affects various strains. *P. gingivalis* is divided into five groups based on *fimA* expression, which encodes fimbriae, a subunit of the *P. gingivalis* filament (Amano et al. 2000). Because different genotypes of *fimA* confer differences in virulence, representative *P. gingivalis* strains and clinical isolates of each type were evaluated. Regardless of the genotype, Kouboku significantly inhibited methyl mercaptan production in *P. gingivalis*. Kouboku elicited similar inhibitory effects on the standard strains and clinical isolates (Figure 1B,C). To confirm the role of *mgl* in methyl mercaptan production, both the wild‐type *P. gingivalis* 33277 strain and the *mgl* deletion mutant were analyzed. Methyl mercaptan production was undetectable in the *mgl* mutant strain regardless of the presence or absence of l‐methionine (Figure 1D).

### 3.2 *mgl* mRNA Expression in *P. gingivalis*

**FIGURE 2:**
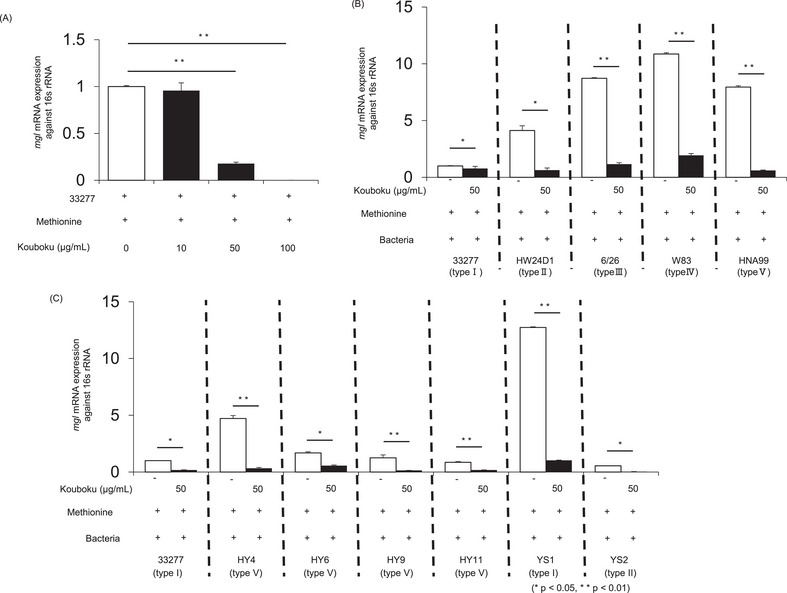
*mgl* mRNA expression in *Porphyromonas gingivalis*. (A) The mRNA expression of *mgl* in *P. ginvialis* 33277 in the presence or absence of Kouboku. *P. gingivalis* cells with different FimA genotypes were studied. Representative standard strains (B) and clinical isolates (C) of *P. gingivalis*. An overnight culture of *P. gingivalis* was pelleted by centrifugation (3000 × *g* for 10 min). The pellet was then washed and resuspended in PBS. The bacterial suspension was adjusted to a density of 1 × 10⁸ bacterial cells/mL, and Kouboku was added (0, 10, 50, or 100 µg/mL). The samples were then cultured in 1.5‐mL tubes and incubated for 10 min at 37°C. *mgl* mRNA expression was quantified after treatment. *p < 0.05, **p < 0.01; Student's *t*‐test.

To determine whether the mRNA expression levels of *mgl* were altered in *P. gingivalis* with different pathogenicities, comparisons were made using *P. gingivalis* with different FimA genotypes. Methyl mercaptan generation is regulated by *mgl*, which encodes METase. To confirm the mechanism by which Kouboku regulates methyl mercaptan production, *mgl* mRNA expression in *P. gingivalis* was determined in the presence or absence of Kouboku. The results showed that the mRNA expression of *mgl* in *P. gingivalis* 33277 was significantly suppressed by Kouboku in a concentration‐dependent manner (Figure 2A). Similar to *P. gingivalis* 33277, a significant suppressive effect was observed against laboratory strains and clinical isolates of *P. gingivalis* (Figure 2B,C).

### 3.3 Biofilm Formation in *P. gingivalis*

**FIGURE 3:**
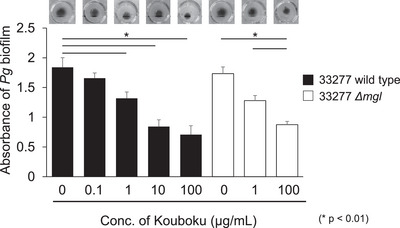
Biofilm formation in *Porphyromonas gingivalis*. The effect of Kouboku on biofilm formation by *P. gingivalis* and *Fusobacterium nucleatum* ATCC10953 co‐cultures was determined. Briefly, overnight cultures of *P. gingivalis* and *F. nucleatum* were pelleted by centrifugation (3,000 ×*g* for 10 min), and the pellet was washed and resuspended in PBS. The bacterial suspension was adjusted to a density of 10⁸ bacterial cells/mL (*P. gingivalis*: *F. nucleatum* = 5:1), and Kouboku (0, 0.1, 1, 10, or 100 µg/mL) and l‐methionine (100 nM) were added to the bacterial suspension. The samples were then cultured in 96‐well plates and incubated for 2 days at 37°C. After washing the plate once with PBS, the biofilm was stained with 1% crystal violet and then washed twice with PBS. The crystal violet was eluted using ethanol, and the absorbance was measured at 450 nm using an ELISA reader (OD405, Varioskan LUX, Thermo Fisher Scientific, Waltham, MA, USA). To quantify biofilm formation, biofilm formation was calculated for each sample and expressed as a ratio relative to the 0 µg/mL Kouboku group (no treatment). *p < 0.01; Student's *t*‐test.

Kouboku did not show strong antimicrobial activity against *P. gingivalis* compared to antibiotics (Ouhara et al. 2015). Several studies have reported the effects of the sub‐MICs of antibiotics on biofilm formation. For instance, sub‐MIC concentrations of antibiotics affect biofilm formation by *Pseudomonas aeruginosa* (Baseri et al. 2022). Next, the effect of Kouboku on biofilm formation in *P. gingivalis* 33277 wild‐type and *F. nucleatum* ATCC10953 cocultures was determined. Kouboku inhibited biofilm formation by *P. gingivalis* 33277 in a concentration‐dependent manner compared to the absence of Kouboku (1 µg/mL, 28.8% inhibition; 10 µg/mL, 54.3% inhibition; 100 µg/mL, 62.0% inhibition; Figure 3). Biofilm formation by the *P. gingivalis* 33277 *mgl* deletion mutant was inhibited by Kouboku in a concentration‐dependent manner compared to conditions without Kouboku treatment (1 µg/mL, 26.0% inhibition; 100 µg/mL, 49.1% inhibition; Figure 3).

### 3.4 Adhesion of *P. gingivalis* Cells to OBA‐9 HGEC Cells

**FIGURE 4:**
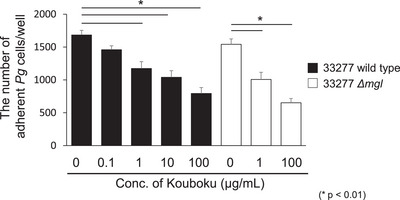
Adhesion of *Porphyromonas gingivalis* to OBA‐9 cells. The adhesion of *P. gingivalis* 33277 wild‐type and *mgl* deletion mutant to OBA‐9 cells was evaluated in the presence and absence of Kouboku. Briefly, the bacterial suspension was adjusted to a density of 1 × 10⁷ bacterial cells/mL, and Kouboku (0, 0.1, 1, 10, or 100 µg/mL) was added to the bacterial suspension. The samples (100 µL) were then incubated for 10 min at 37°C, added to 96‐well plates containing OBA‐9 cells (10⁵ cells per well), and incubated for 2 h at 37°C. Samples cultured in 96‐well plates were washed thrice with PBS, and 0.25% trypsin was added to remove adherent bacterial cells. After adding 100 µL of PBS, the bacterial suspension was diluted appropriately, plated on TSA agar, and incubated for 3 days at 37°C. The colonies were counted as adhesive bacterial cells. *p < 0.01; Student's *t*‐test.

Because the adhesion of bacteria to host cells triggers *P. gingivalis* colonization and VSC production, the adhesion of *P. gingivalis* 33277 to OBA‐9 cells in the presence or absence of Kouboku was assessed. The number of wild‐type *P. gingivalis* 33277 adhering to OBA‐9 cells with the addition of Kouboku was significantly lower than that without Kouboku (1 µg/mL, 30.3% suppression; 10 µg/mL, 38.2% suppression; 100 µg/mL, 52.9% suppression; Figure 4). As for *P. gingivalis* 33277 *mgl* deletion mutant, the adherent bacterial cell to host cells was also inhibited by Kouboku in a concentration‐dependent manner compared to the absence of Kouboku (1 µg/mL, 34.7% inhibition; 100 µg/mL, 59.5% inhibition; Figure 3)

### 3.5 Synergistic Antibacterial Effect of the Active Ingredients of Kouboku (Honokiol and Magnolol) and Antibiotics

To determine the synergistic antibacterial effects of the active ingredients of Kouboku (honokiol and magnolol) and various antibiotics, *P. gingivalis* 33277 was cultured in the presence or absence of honokiol and magnolol with antibiotics for three days. The MIC of antibiotics against *P. gingivalis* varied in the presence of honokiol and magnolol. Specifically, the MICs of the antibiotics in the presence of honokiol were lower than those in the absence of honokiol (ampicillin, 75% suppression; gentamycin, 75% suppression; erythromycin, 93.8% suppression; tetracycline, 50% suppression). The same trend was observed for magnolol (ampicillin, 87.5% suppression; gentamicin, 87.5% suppression; erythromycin, 96.9% suppression; tetracycline, 50% suppression) (Table 1). Therefore, the augmentation of the antibacterial effect of the tested antibiotics was attributed to synergy with Kouboku (honokiol and magnolol).

**TABLE 1:** Synergistic antibacterial effects of the active ingredients of Kouboku (honokiol and magnolol) and antibiotics.

	MICs of antibiotics (µg/mL)					
Strain	Ampicillin	Gentamycin	Erythromycin	Tetracyclin	Honokiol	Magnolo
*Porphyromonas gingivalis* 33277	16	16	4	0.5	8	8
*P. gingivalis* 33277 (Honokiol 5 µg/mL)	4	4	0.25	0.25	—	—
*P. gingivalis* 33277 (Magnolol 5 µg/mL)	2	2	0.125	0.25	—	—

*Note*: Measurement of minimum inhibitory concentrations (MIC) of antibiotics. Briefly, 2‐day cultures of *P. gingivalis* 33277 were pre‐diluted to an optical density at 660 nm (OD_660_) of 0.1, and 10 µL of this diluted culture was added to a 96‐well plate containing the appropriate culture medium. The plate was then incubated for 3 days at 37°C under anaerobic conditions. To measure the MIC, culture media containing the antibiotic solutions, magnolol or honokiol, were serially 1:2‐diluted to final concentrations of 1024 µg/mL to 0.03 µg/mL. MIC was determined visually as the lack of turbidity after 72 h of treatment. The MIC of the antibiotics varied in the presence of Kouboku. The MIC of the antibiotics in the presence of honokiol was lower than that in its absence, and the same trend was observed for magnolol.

### 3.6 mRNA Expression of fimA in *P. gingivalis*

**FIGURE 5:**
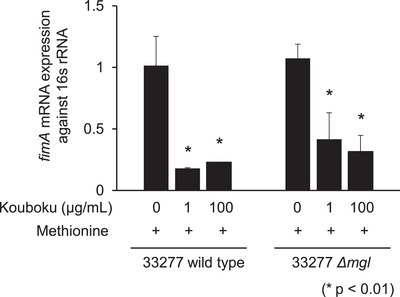
*mgl* mRNA expression in *Porphyromonas gingivalis*. (A) The mRNA expression of *fimA* in *P. gingivalis* 33277 wild type and *mgl* deletion mutant in the presence or absence of Kouboku. The bacterial suspension was adjusted to a density of 1 × 10⁸ bacterial cells/mL, and Kouboku was added (0, 1, or 100 µg/mL). The samples were then cultured in 1.5‐mL tubes and incubated for 10 min at 37°C. The expression of *fimA* mRNA was quantified after treatment. *p < 0.01; Student's *t*‐test.

To investigate the mechanism by which Kouboku affects biofilm formation and adhesion to OBA‐9 cells, the expression of *fimA* mRNA was quantified in both the wild‐type and *mgl*‐deletion mutant strains. Kouboku treatment suppressed *fimA* expression in a concentration‐dependent manner in both strains compared to the untreated controls (wild type: 77.1% suppression; *mgl* deletion mutant: 68.4% suppression; Figure 5). These findings showed that the target molecule of Kouboku for biofilm formation and cellular attachment was not *mgl* but *fimA*.

## 4 Discussion

In the present study, Kouboku showed inhibitory effects in *mgl*‐regulated methyl mercaptan production and *P. gingivalis* biofilm formation and adhesion to OBA‐9 HGECs. Similar to previous reports, the inhibitory effect of Kouboku on methyl mercaptan production in standard *P. gingivalis* strains and clinical isolates was observed at both the mRNA and gas production levels. In addition, Kouboku inhibited the ability of *P. gingivalis* to form biofilms and adhere to host cells. Moreover, the addition of honokiol and magnolol, the active components of Kouboku, to antibiotics decreased their MICs. These findings indicate that Kouboku is more effective against bacteria as a pathogenic factor than against bacteria alone.

*P. gingivalis* fimbriae seem to participate in interactions between the bacterium and the host and with other bacteria (Hajishengallis et al. 2012). This pathogen expresses two distinct fimbria molecules on its cell surface. One is composed of a subunit protein named FimA (or fimbrillin) and termed long or long fimbriae. The other consists of a subunit protein named Mfa, which is encoded by *mfa1* and is termed short, minor, or Mfa fimbriae (Amano et al. 2000). FimA is encoded by *fimA* and occurs as a single copy on the *P. gingivalis* chromosome. Based on nucleotide sequence variations, *fimA* has been classified into six types (I, Ib, II, III, IV, and V) (Fujiwara et al. 1993; Nakagawa et al. 2002). The pathogenicity conferred by various FimA genotypes has also been evaluated in animal models. FimA genotypes II, Ib, and IV cause stronger infectious symptoms and inflammatory changes than strains harboring genotypes I and III (Amano et al. 2004; Nakano et al. 2004). In particular, type II FimA is associated with the pathogenesis of periodontitis (Kato et al. 2007). Thus, because the pathogenicity of *P. gingivalis* varies among different types of fimbriae, we examined the relationship between the fimbriae type and methyl mercaptan production. Regardless of the fimbriae type, Kouboku effectively suppressed methyl mercaptan production in all *P. gingivalis* standard strains and clinical isolates (Figure 1). Similarly, we also compared *mgl* expression and FimA genotype. Kouboku suppressed *mgl* mRNA expression in all *P. gingivalis* standard strains and clinical isolates, regardless of the FimA type (Figure 2).

*P. gingivalis* disrupts host barrier systems and induces inflammation using pathogenic factors such as lipopolysaccharides, proteases, and fimbriae (Xu et al. 2020). The cysteine proteases derived from *P. gingivalis*, including the gingipains Rgp and Kgp, are thought to be neurotoxic and involved in the exacerbation of Alzheimer's disease by interfering with normal neural mechanisms. Gingipains can reduce the concentrations of cytokines in cell culture systems and digest and inactivate TNF‐α (Calkins et al. 1998). *P. gingivalis* also exerts substantial proteolytic effects on lipoproteins (Lönn et al. 2018). Thus, we believe that gingipain‐induced toxicity is due to its proteolytic activity. Methyl mercaptan is also regarded as a virulence factor because it injures host cells. Furthermore, intraperitoneal injection of *mgl*‐deficient *P. gingivalis* mutants into mice led to a higher survival rate than injection of wild‐type *P. gingivalis* (Yoshimura et al. 2000). Methyl mercaptan is produced from l‐methionine through the enzymatic action of METase, which catalyzes the α, γ elimination of l‐methionine to produce α‐ketobutyrate, methyl mercaptan, and ammonia (Yoshimura et al. 2000). Gingipain may act as a virulence factor by affecting the methionine metabolic pathway and promoting methyl mercaptan production.

Biofilm formation is a complex process involving reversible and irreversible bacterial attachment, microcolony formation, formation of a stable three‐dimensional structure, and dispersion (Kuboniwa and Lamont 2010). The roles of the long and short fimbriae of *P. gingivalis* in biofilm formation are likely different. The effects of a set of fimbriae and gingipains on homotypic biofilm formation have been previously examined using deficient mutants (Kuboniwa et al. 2009). Their results suggest that long fimbriae promote initial biofilm formation and exert a restraining effect on biofilm maturation. In contrast, short fimbriae and Kgp play suppressive and regulatory roles, respectively, during biofilm development. Furthermore, Rgp likely controls the microcolony morphology and biovolume. Collectively, these molecules appear to act in a coordinated manner to regulate the development of mature biofilms (Enersen et al. 2013).

In the present study, Kouboku inhibited *P. gingivalis* biofilm formation when co‐cultured with *F. nucleatum* (Figure 3). To support the proposed mechanism of the effect of Kouboku on biofilm formation and cellular adhesion, we measured the mRNA expression of *fimA* in *P. gingivalis*. We found that *fimA* expression was suppressed in the *P. gingivalis* 33277 wild‐type strain (Figure 5). The concentration of Kouboku that suppressed *fimA* expression (1 or 100 µg/mL) was much lower than the MIC (256 µg/mL). Thus, the observed inhibitory effects on biofilm formation and cellular adhesion to host cells were unlikely to be due to bacterial cytotoxicity. Instead, Kouboku may suppress the expression of adhesion factors, such as *P. gingivalis* fimbriae, thereby inhibiting biofilm formation. Further studies, including 3D analysis of biofilm formation using electron and fluorescence microscopy, are required to test this hypothesis.

The major fimbriae of *P. gingivalis*, encoded by *fimA*, are involved in the adhesion to epithelial cells and subsequent signaling events leading to invasion (Umeda et al. 2006). In this study, Kouboku inhibited the adhesion of *P. gingivalis* to epithelial cells (Figure 4). Kouboku treatment also suppressed methyl mercaptan production and *mgl* mRNA expression, regardless of the *P. gingivalis* FimA genotype. Kouboku inhibited both biofilm formation and cellular adhesion to host cells. These findings suggest that Kouboku may reduce the cytotoxicity of *P. gingivalis* by targeting cell surface adhesion factors such as fimbriae. Moreover, Kouboku not only suppressed methyl mercaptan production via downregulation of *mgl* but also reduced *fimA* expression in both the *P. gingivalis* 33277 wild‐type strain and the *mgl* deletion mutant. These results indicate that the suppressive effect of Kouboku on biofilm formation and cellular adhesion to host cells is not mediated by *mgl*.

Magnolol and honokiol are known to be the active ingredients of Kouboku. It has been reported that gum containing magnolol and honokiol reduces the number of bacteria in the saliva (Greenberg et al. 2007). In particular, magnolol has been shown to balance the intestinal microbiota in ulcerative colitis and has antimicrobial activity against floating *Streptococcus mutans* and *S. mutans* biofilms (Sakaue et al. 2016; Xie et al. 2022). Based on the results of this and previous studies, it is likely that the active ingredients in Kouboku, such as magnolol and honokiol, exert antimicrobial activity by acting on bacterial cells and biofilms. In this study, when the MIC of each antimicrobial agent was measured after the addition of magnolol and honokiol at concentrations that did not show antimicrobial activity, a decrease in MIC was observed (Table 1). Generally, antimicrobial agents are resistant or inactivated when used at concentrations below their MIC; therefore, it is important to maintain effective concentrations in accordance with Antimicrobial Resistance (AMR). In the present study, Kouboku inhibited both biofilm formation and cell adhesion ability (Figures 3 and 4). However, since both effects were observed at concentrations lower than when antimicrobial effects were observed, it might also be due to the control of adhesion factors on the surface layer of the bacteria, in addition to *mgl* expression. Our previous report showed that the MIC of Kouboku was 256 µg/mL (Ouhara et al. 2015). Sub‐MIC concentrations of Kouboku did not affect the growth of *P. gingivalis*. Therefore, although a more detailed investigation is required, our observations suggest that the antibacterial effect of Kouboku and the mechanism of suppressing *mgl* and *fimA* mRNA expression may differ. Magnolol and honokiol may have bound to the surface layer of the bacteria or taken up by the bacterial cells to exert their effects, thereby affecting the adhesion of bacteria to each other and to their host cells. A more detailed study is required to confirm this hypothesis.

In conclusion, magnolol and honokiol may bind to the surface layer of bacteria and be taken up by bacterial cells to exert their effects, affecting the adhesion of bacteria to each other and their host cells. As Kouboku has shown a broad inhibitory effect on halitosis gas production and *P. gingivalis*, it is expected to be clinically applicable; therefore, further studies are required. Further clinical studies using more types of *P. gingivalis* isolates from patients with periodontal disease are needed to clarify the effect of Kouboku on the amount of methyl mercaptan in the oral cavity of these patients.

## Conflicts of Interest

The authors declare no conflicts of interest.

### Peer Review

The peer review history for this article is available at https://publons.com/publon/10.1111/omi.12493

## Data Availability

All data generated or analyzed during this study are included in the published article.
